# In-vitro function of upstream visfatin polymorphisms that are associated with adverse cardiometabolic parameters in obese children

**DOI:** 10.1186/s12864-016-3315-9

**Published:** 2016-11-25

**Authors:** Delicia Shu Qin Ooi, Siong Gim Ong, Chew Kiat Heng, Kah Yin Loke, Yung Seng Lee

**Affiliations:** 1 Department of Paediatrics, Yong Loo Lin School of Medicine, National University of Singapore, Singapore, Singapore; 2 Division of Paediatric Endocrinology and Diabetes, Khoo Teck Puat-National University Children’s Medical Institute, National University Health System, Singapore, Singapore; 3 Singapore Institute for Clinical Sciences, Agency of Science, Technology and Research, Singapore, Singapore; 4 Department of Paediatrics, Yong Loo Lin School of Medicine, NUHS tower block, Level 12, 1E Kent Ridge Road, Singapore, 119228 Singapore

**Keywords:** Childhood obesity, Visfatin promoter, Luciferase assay, Polymorphisms, Variants

## Abstract

**Background:**

Visfatin is an adipokine associated with glucose and lipid metabolism. We previously reported two visfatin upstream single nucleotide polymorphisms (SNPs), c.-3187G > A (rs11977021) and c.-1537C > T (rs61330082), which were in perfect linkage disequilibrium, in a Singaporean cohort of severely obese children and are associated with visfatin level and adverse cardiometabolic parameters. We aim to functionally characterize the effect of c.-3187G > A and c.-1537C > T SNPs on basal transcriptional activity.

**Methods:**

A 1.6 kb and 3.7 kb upstream promoter region of the visfatin gene was amplified by polymerase chain reaction and separately cloned into luciferase reporter vectors. Successful clones were transfected into human embryonic kidney (HEK293T) and human breast carcinoma (MCF7) cells and in-vitro dual-luciferase assay was performed. ﻿Electrophoretic mobility shift assay (﻿﻿EMSA) was also conducted to examine the binding affinity between transcription factors and visfatin promoter sequences.

**Results:**

Variant promoter with only c.-1537C > T SNP did not show a change in transcriptional activity as compared to the wild type. However, variant promoter with both c.-3187G > A and c.-1537C > T SNPs showed a statistically significant increase of 1.41 fold (*p* < 0.01) in transcriptional activity. The longer 3.7kbp visfatin promoter sequence was also shown to have significantly higher transcriptional activity (*p* < 0.05) as compared to the shorter 1.6kbp visfatin promoter. Both c.-3187G > A and c.-1537C > T variants showed an increased binding with nuclear protein.

**Discussion and conclusions:**

We have demonstrated for the first time that visfatin variant promoter with both c.-3187G > A and c.-1537C > T SNPs result in an increase in transcriptional activity. This supports our previous finding and postulation that these SNPs contribute to elevated visfatin levels which may mediate higher triglyceride levels, severe systolic blood pressure and severe hypertension in obese children. These SNPs may co-operatively affect enhancer or silencer function to regulate transcriptional activity. In conclusion, this study shows that upstream visfatin SNPs could potentially affect phenotypic outcome in obese children through alteration of circulating visfatin level.

**Electronic supplementary material:**

The online version of this article (doi:10.1186/s12864-016-3315-9) contains supplementary material, which is available to authorized users.

## Background

Visfatin is an adipokine secreted by adipose tissues [1] and plasma visfatin was shown to correlate with obesity and inflammation [2, 3]. The human visfatin gene (NC_000007.13) is mapped at 7q22.3 on the long arm of chromosome 7, it spans at an approximate length of 34.7kbp and consists of 11 exons and 10 introns [4]. The upstream promoter sequence of visfatin contains multiple binding sites for transcription factors such as nuclear factor-1 (NF-1), activator protein (AP-1 and AP-2), specificity protein-1 (SP-1) and glucocorticoid receptor (GR) [4]. Studies have identified several common upstream single nucleotide polymorphisms (SNPs) found to be associated with inflammation and parameters of glucose and lipid metabolism [5–8]. Among the reported upstream SNPs, the c.-1537C > T SNP (rs61330082) has been shown to cause a reduction in the transcriptional activity of luciferase reporter assay [9, 10]. However, another study by Tokunaga et al. found no difference in reporter gene expression between C and T allele of c.-1537C > T SNP [11]. Our group has previously reported perfect linkage disequilibrium between rs61330082 and another SNP, c.-3187C > T (rs11977021) which was found to be significantly associated with total plasma cholesterol and LDL-cholesterol levels in French-Canadian subjects [5]. These two SNPs were also shown to be associated with obesity, severe systolic blood pressure, severe hypertension, plasma visfatin and triglyceride levels in our cohort of local severely obese children [12]. We hypothesized that the two upstream promoter SNPs (rs61330082 and rs11977021) may have a functional effect on the transcriptional activity of the visfatin gene.

## Methods

### PCR, cloning and construction of visfatin plasmid

Polymerase chain reaction (PCR) was performed to amplify the upstream promoter region of visfatin. The DNA samples of unrelated obese subjects identified to be homozygous for the respective wild-type and variant alleles of the 2 SNPs (rs61330082 and rs11977021) at perfect linkage disequilibrium were used for PCR [12]. The primers used to amplify an amplicon of 3.7kbp upstream from start codon ATG of visfatin gene were 5’-GTA CTC GAG GCC GGT TAG GAG AGT GCA GCA CAG-3’ (forward) and 5’- GAA CTA AGA TCT CTC GGG CCG GAG GAC AGG GGC -3’(reverse). The primers used to amplify an amplicon of 1.6kbp upstream were 5’-GAC CTC GAG TGT TTC AAA CCT CGT TGC T-3’ (forward) and 5’- GAA CTA AGA TCT CTC GGG CCG GAG GAC AGG GGC -3’(reverse). The visfatin insert was then cloned into pGL4.10 luciferase reporter vector [Promega, USA] through BglII and XhoI restriction enzyme digestion. Successful clones were obtained and random variants in the clones were rectified by site-directed mutagenesis. The sequences of all plasmids were verified by DNA sequencing to ensure no other mutations were present (Additional files 1 and 2: Figures S1 and S2).

### Cell transfection and luciferase assays

Human embryonic kidney cells, HEK293 and human breast cancer cells (MCF-7) were grown to 90% confluency. The seeding density of the cells was ~5 × 10^4^ cells/well and they were pre-cultured in 24-well tissue culture plate for 24 h.

Cells were co-transfected with 1 ng/μl of visfatin pGL4.10 luciferase plasmid and 0.025 ng/μl of Renilla luciferase reporter vector using 0.03 μl /μl of Lipofectamine 2000 [Invitrogen, USA]. The transfected cells were incubated at 37 °C with 5% CO_2_ overnight for 24 h before they were used for luciferase assay study. The functionality of the visfatin insert was determined by dual-luciferase reporter assay [Promega, USA] according to manufacturer’s instructions. The assay for each visfatin insert was performed in triplicates and the experiment was performed thrice. All the 3 transfection experiments showed similar fold-change. However, due to the variable transfection efficiency, we presented the transfection results as the mean and SD of the triplicates in one experiment setting. The results for other 2 biological replicates are shown in Additional file 3: Figure S3.

### Prediction of the functional consequences of the variants

We investigated whether the promoter variants have any effect on putative transcription factor binding sites within the visfatin gene using PROMO. The parameters used in ALGGEN-PROMO program were “only human factors” and “only human sites”. (http://alggen.lsi.upc.es/cgi-bin/promo_v3/promo/promoinit.cgi?dirDB=TF_8.3, last accessed on 30^th^ May 2014) [13, 14]. MotifMap was also used to check the nucleotide sequence of the transcription factor binding sites.

### Electrophoretic mobility shift assay (EMSA)

We extracted nuclear protein from cultured MCF7 cells using the NE-PER nuclear and cytoplasmic extraction kit according to manufacturer’s instructions [ThermoFisher Scientific, USA]. Biotin-labeled probes of the visfatin promoter sequence containing the respective c.-3187G > A and c.-1537C > T variant were synthesized [Integrated DNA technologies, USA]. The probes represent a short section (11 bases) of the visfatin promoter sequence and were designed to include 5 bases before and after the respective SNP. The probe sequences are c.-3187G: 5’-ACTGA**G**GTCAA-3’; c.-3187A: 5’-ACTGA**A**GTCAA-3’; c.-1537C: 5’- AGTGC**C**TGGTG-3’; C.-1537 T: 5’- AGTGC**T**TGGTG-3’. The biotin-labeled probes were incubated with or without the nuclear protein extract and in the presence or absence of unlabeled probes. EMSA was carried out using the LightShift chemiluminescent EMSA kit according to manufacturer’s instructions [ThermoFisher Scientific, USA]. The experiment was performed twice (Additional file 4: Figure S4) and the density of each band was quantified by GeneTool [Syngene, Cambridge, England].

### Statistical analyses

Data are expressed as mean ± standard deviation (SD). The difference in luciferase activity between different visfatin promoter sequences was analyzed by the Student’s *t*-test. Paired sample *t*-test was used to analyze the difference in EMSA band intensity between wild-type and variant promoter sequence [SPSS Version 22, USA].

## Results

### Transcription factor binding sites in visfatin promoter sequence

**Fig. 1:**
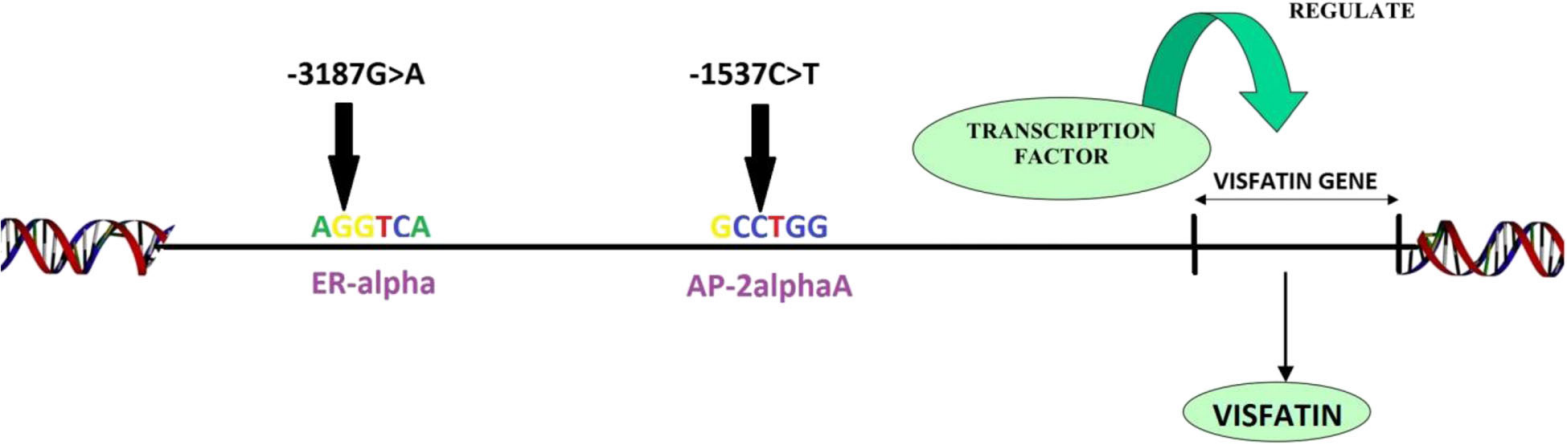
Promoter region of visfatin gene. The sequence representing the ER-alpha and AP-2alphaA transcription factor binding sites located respectively at −3187 and −1537 of the visfatin promoter region as predicted by PROMO are shown

We used the transcription factor binding site prediction program PROMO to predict any alteration in transcription factor binding affinity in visfatin promoter sequence previously found to contain single nucleotide polymorphisms (SNPs), rs61330082 (c.-3187G > A) and rs11977021 (c.-1537C > T). PROMO predicted the alteration of the transcription factor estrogen receptor-alpha (ER-alpha) binding site by c.-3187G > A variant while c.-1537C > T variant was predicted to alter activating protein-2alphaA (AP-2alphaA) transcription factor binding site as shown in Fig. 1.

### Comparison of transcriptional activity between visfatin promoter variants

**Fig. 2:**
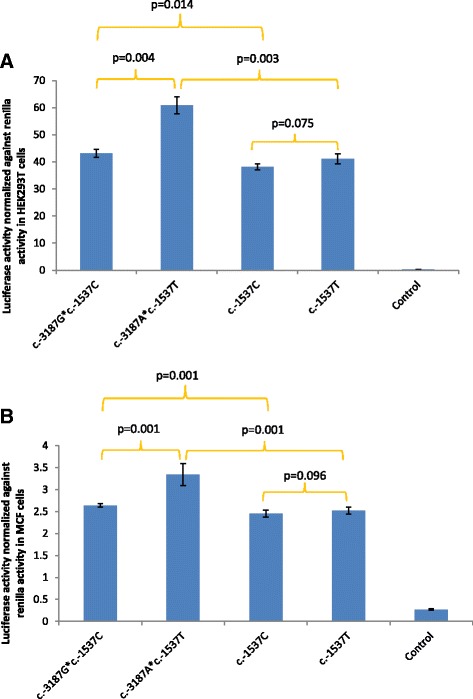
Basal transcriptional activity of visfatin promoter variants as measured in-vitro by luciferase assay. The average relative transcriptional activities of the visfatin promoter variants in **a** HEK293T and **b** MCF7 cells are indicated in shaded bars and the standard deviation (SD) is represented by the error bars. The *p*-values for the comparison of in-vitro transcriptional activity between the promoter sequences are stated. c.-3187*c.-1537 indicates a 3.7kbp promoter sequence containing both c.-3187 and c.-1537 SNPs, while c.-1537 indicates a 1.6 kbp promoter sequence containing only c.-1537 SNP

The basal transcriptional activity was measured as the in-vitro luciferase activity of the different visfatin promoter sequences in transiently transfected MCF7 and HEK293T cells as shown in Fig. 2. The transcriptional activity of the variant 3.7kbp promoter sequence containing both c.-3187G > A and c.-1537C > T SNPs was 1.27 and 1.41 fold higher than that of wild-type 3.7kbp promoter in MCF7 and HEK293T cells respectively. The variant 1.6kbp visfatin promoter sequence containing only c.-1537C > T SNP showed no significant change in luciferase activity as compared to wild-type 1.6kbp promoter in both MCF7 and HEK293T cells. The transcriptional activity of the wild-type 3.7kbp promoter sequence was 1.08 and 1.13 fold (*p* = 0.014) higher than that of wild-type 1.6kbp promoter in MCF7 and HEK293T cells respectively. The transcriptional activity of the variant 3.7kbp promoter sequence was 1.27 and 1.48 fold higher than that of variant 1.6kbp promoter in MCF7 and HEK293T cells respectively. The luciferase assay was performed in triplicates (*n* = 3) for each promoter sequence and a minimal amount of luciferase activity (0.21 ± 0.03 and 0.27 ± 0.02) was detected in HEK293T and MCF cells ﻿transfected with control pGL4.10 luciferase plasmid.

### Binding of transcription factors to visfatin variant promoter sequence

**Fig. 3:**
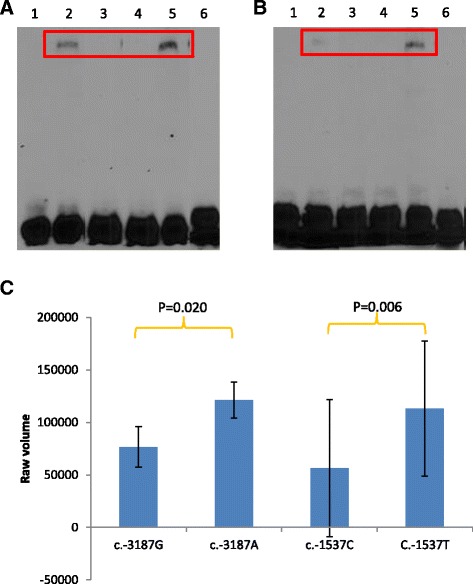
Binding of transcription factors to visfatin promoter variants as measured in-vitro by EMSA. **a** EMSA for c.-3187G > A SNP only, **b** EMSA for c.-1537C > T SNP only, **c** Assessment of binding between visfatin SNPs and nuclear protein, the EMSA band density are indicated in shaded bars and the standard deviation (SD) between 2 EMSA experiments is represented by the error bars. (1): Wild-type sequence without nuclear protein, (2): Wild-type sequence with nuclear protein, (3): Wild-type sequence with nuclear protein and unlabeled probe, (4): Variant sequence without nuclear protein, (5): Variant sequence with nuclear protein, (6): Variant sequence with nuclear protein and unlabeled probe

EMSA data showed that the variant alleles in the respective c.-3187G > A and c.-1537C > T SNPs have a significant increased binding with nuclear protein (Fig. 3a, b). The binding between the visfatin variant promoter sequence and nuclear protein is assessed by densitometry and the large standard deviation values were due to comparison between 2 separate EMSA experiments (Fig. 3c).

## Discussion and conclusion

In this study, we functionally characterized two upstream visfatin promoter SNPs, c.-3187G > A (rs11977021) and c.-1537C > T (rs61330082), that were previously detected in the upstream promoter region of visfatin gene in our cohort of severely obese children [12]. Through the use of the transcription factor binding site prediction program PROMO, the c.-3187G > A and c.-1537C > T SNPs in the visfatin promoter were predicted to alter the ER-alpha and AP-2alphaA transcription factor binding sites respectively (Fig. 1). Wang et al. reported that the c.-1537C > T SNP altered promoter binding to AP-1 [9] but the study did not examine the promoter binding affinity to AP-2alpha2 which can also be a likely transcription factor that is affected by the c.-1537C > T SNP due to the homology between the promoter sequence and AP-2alphaA as predicted by PROMO.

The 3.7kbp visfatin variant promoter sequence containing both c.-3187G > A and c.-1537C > T SNPs was shown to have a significantly higher luciferase activity than that of the wild-type 3.7kbp promoter (Fig. 2). This indicated that the 3.7kbp variant promoter has an increased transcriptional activity and this is in accordance with our previous finding that the two SNPs were associated with elevated serum visfatin (6.17 ± 0.76 ng/ml vs. 3.92 ± 0.44 ng/ml) in our cohort of severely obese children [12].

In addition, we examined the transcriptional activity of the 1.6kbp variant visfatin promoter containing only c.-1537C > T SNP and found that there is no significant difference in transcriptional activity as compared to wild-type 1.6kbp promoter (Fig. 2). Our finding is similar to that reported by Tokunaga et al. who also found no difference in transcriptional activity between variant 1.6kbp visfatin promoter containing c.-1537C > T SNP and wild-type 1.6kbp promoter [11]. However, this differs from the reports by Wang et al. and Ye et al. who showed that c.-1537C > T SNP was associated with lower transcriptional activity [9, 10]. This difference may be attributed to the length of promoter sequence included in the luciferase reporter assay. While Wang et al. and Ye et al. have included ~216 bp and ~147 bp of the visfatin promoter sequence respectively [9, 10], our study has included a ~1.6kbp promoter sequence upstream of the ATG start codon similar to that in the paper by Tokunaga et al. [11]. The putative upstream promoter region of visfatin gene has been shown to contain multiple transcription factor binding sites [4]. Hence, a full promoter sequence from the ATG start site may account for the effect of other transcription factors on the transcriptional activity of the promoter.

The longer 3.7kbp wild-type visfatin promoter was found to have a significantly higher transcriptional activity as compared to the shorter 1.6kbp wild-type promoter. Moreover, the longer 3.7kbp variant promoter was also found to exhibit a higher transcriptional activity as compared to the shorter 1.6kbp variant promoter (Fig. 2). Though the in-vivo significance of this finding is unclear, we speculate that it might imply that the longer 3.7kbp region is more representative of the putative promoter. The higher transcriptional activity of the wild-type 3.7kbp promoter may also contribute in part to the accentuated in-vitro activity of 3.7kbp variant promoter. In addition, the two SNPs, c.-3187G > A and c.-1537C > T, at perfect linkage disequilibrium may have combinatorial effect on the transcriptional activity of the promoter as it has been shown that variants at linkage disequilibrium located at multiple enhancer sites may cooperatively dictate transcript expression [15]. Therefore, unlike previous studies which only examine the effect of c.-1537C > T SNP, our study takes into account distal promoter variants that may have combinatorial effect on the transcriptional activity of the promoter.

Both c.-3187G > A and c.-1537C > T SNPs have been reported as expression quantitative trait loci (eQTLs) in left ventricular heart tissue with trends of decreasing expression as the genotype changes from homozygous wild-type to heterozygous to homozygous variant [16]. However, our results are contrary to that of the reported eQTLs and this may be possibly due to these SNPs/reported eQTLs having different effects on different cell types, resulting in the difference in expression level. Hence, the choice of cell model should be taken into consideration when examining the effect of SNPs on expression level.

Data from our EMSA experiments indicated an increased binding of nuclear protein to the variant A and T alleles of c.-3187G > A and c.-1537C > T SNPs respectively (Fig. 3). This supports the increased transcriptional activity observed in the 3.7kbp visfatin variant promoter sequence (Fig. 2). Although c.-1537C > T SNP also showed an increase in nuclear protein binding, it did not result in an increase in transcriptional activity. This may be due to other upstream SNPs at different regions of the promoter sequence which may cooperatively affect enhancer or silencer function to regulate transcriptional activity.

Since c.-3187G > A does not exist in solitary without c.-1537C > T in our obese children cohort, we did not examine the effect of c.-3187G > A SNP alone on the transcriptional activity. Hence, we could not determine if both SNPs are needed to work cooperatively in order to exert an effect on transcriptional activity. In addition, we did not examine the specific transcription factor that is affected by the c.-3187G > A and c.-1537C > T SNPs respectively. Hence, we are not able to determine if the SNPs have affected binding with ER-alpha and AP-2alphaA transcription factors as predicted by PROMO.

In conclusion, we have demonstrated for the first time that visfatin variant promoter with both c.-3187G > A and c.-1537C > T SNPs result in increased in-vitro transcriptional activity. This supports our previous finding and postulation that these SNPs contribute to elevated visfatin levels which mediate higher triglyceride levels, severe systolic blood pressure and severe hypertension in obese children. This study supports the role of these upstream visfatin SNPs which could potentially affect phenotypic outcome in obese children through alteration of circulating visfatin level.

## Additional files

Additional file 1: Supplementary Figure 1. Plasmid sequence for wild-type visfatin promoter. (DOCX 867 kb)
Additional file 2: Supplementary Figure 2. Plasmid sequence for variant visfatin promoter. (DOCX 1313 kb)
Additional file 3: Supplementary Figure 3. ﻿Biological replicates for luciferase assays﻿. (DOCX 233 kb)
Additional file 4: Biological replicate for EMSA. (DOCX 372 kb)

## Abbreviations

AP-1/AP-2: Activator protein-1/2
AP-2alphaA: Activating protein-2alphaA
EMSA: Electrophoretic Mobility Shift Assay
eQTL: Expression quantitative trait loci
ER-alpha: Estrogen receptor-alpha
GR: Glucocorticoid receptor
HEK: Human embryonic kidney
NF-1: Nuclear factor-1
PCR: Polymerase chain reaction
SD: Standard deviation
SNP: Single nucleotide polymorphism
SP-1: Specificity protein-1
