# Supplementary figures and images for: In-vitro function of upstream visfatin polymorphisms that are associated with adverse cardiometabolic parameters in obese children
Source: BMC Genomics. 2016 Nov 25;17:974. doi: 10.1186/s12864-016-3315-9 (PMC5124300; doi:10.1186/s12864-016-3315-9)

**Supplementary Figure 1**


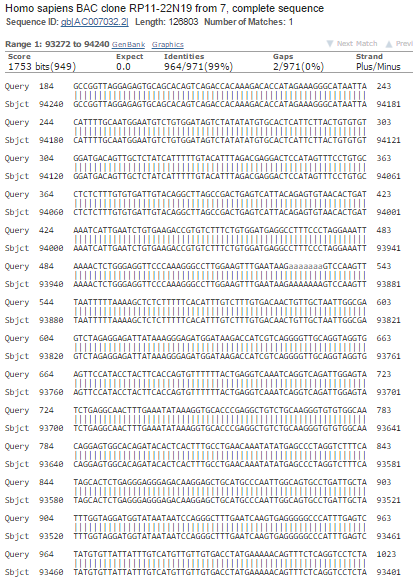


-3187G


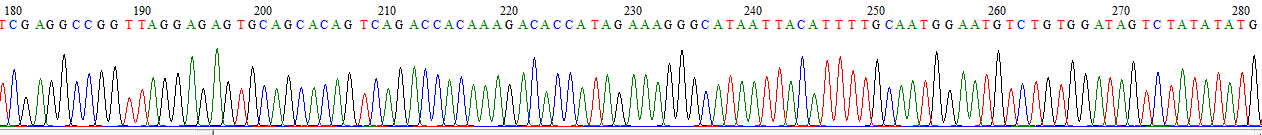


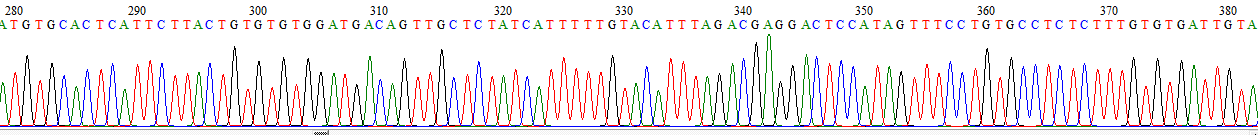


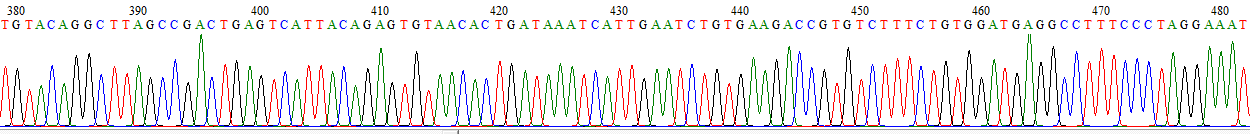


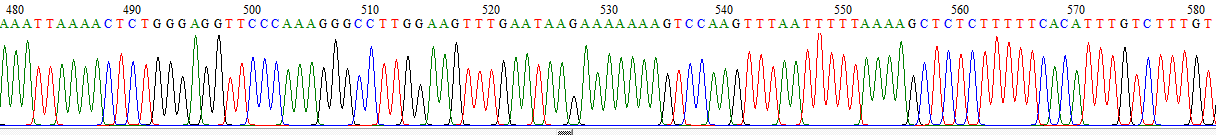


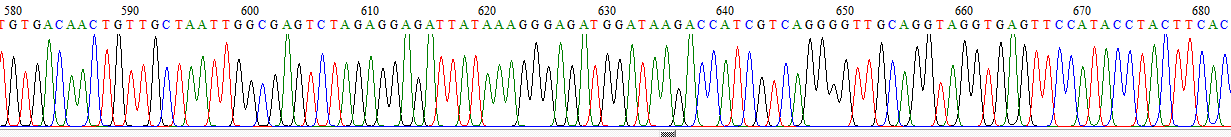


-3187G


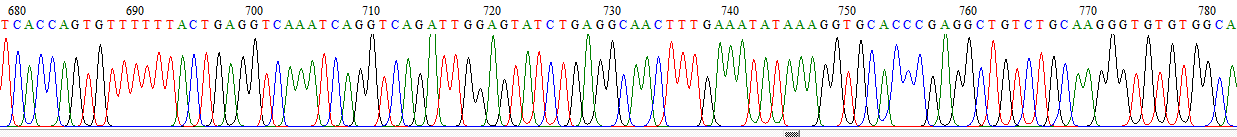


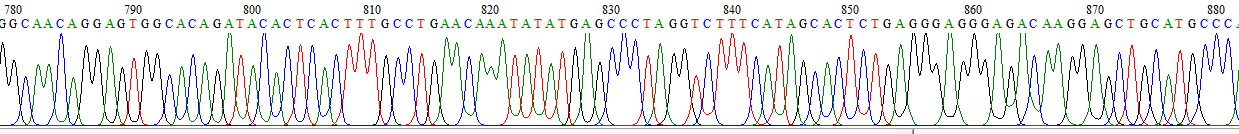


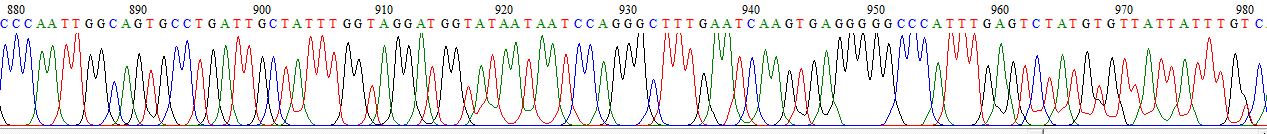


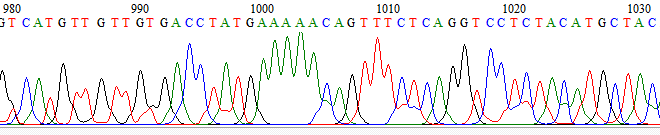


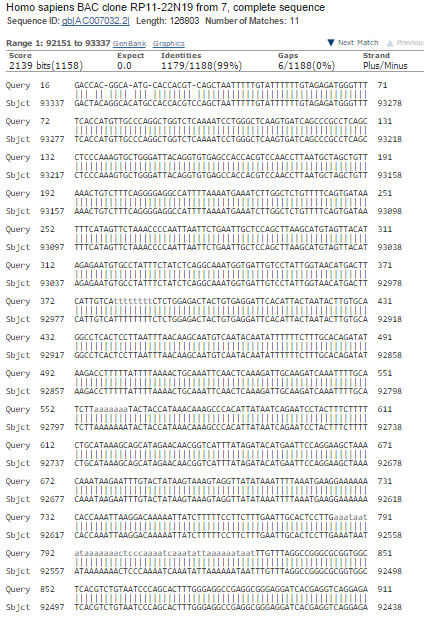


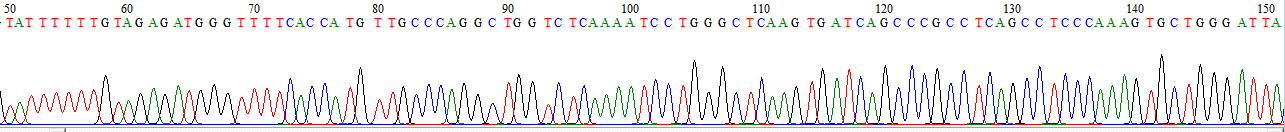


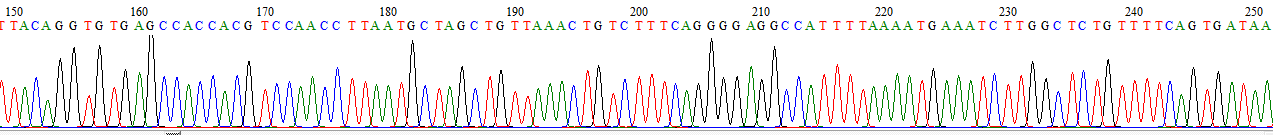


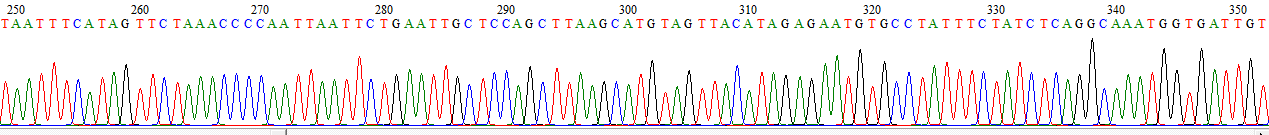


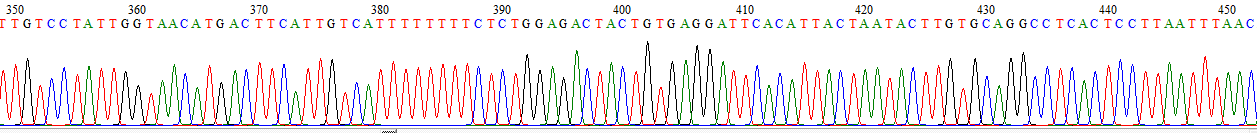


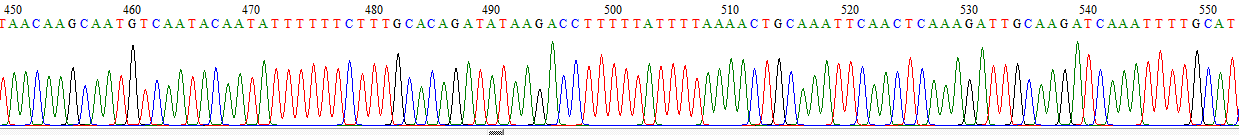


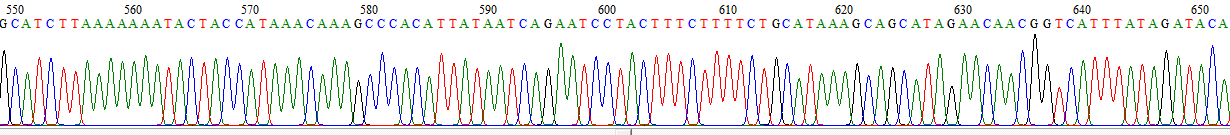


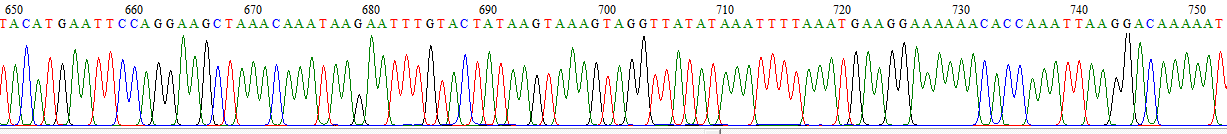


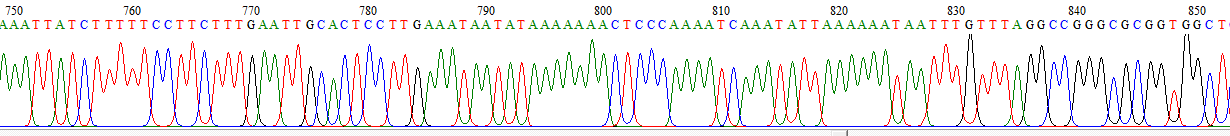


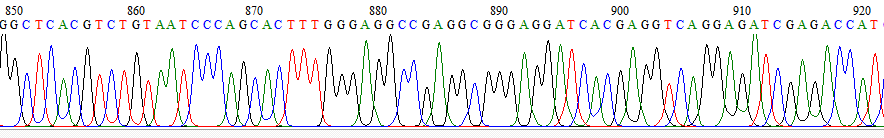


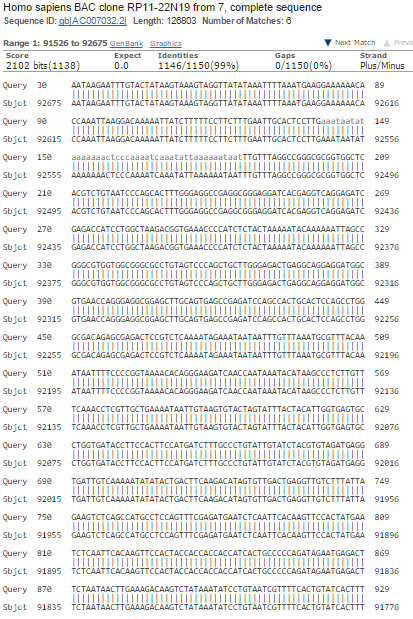


-1537C


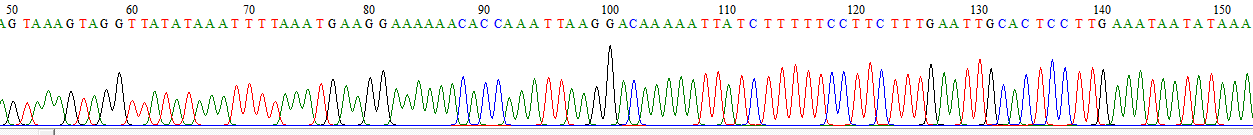


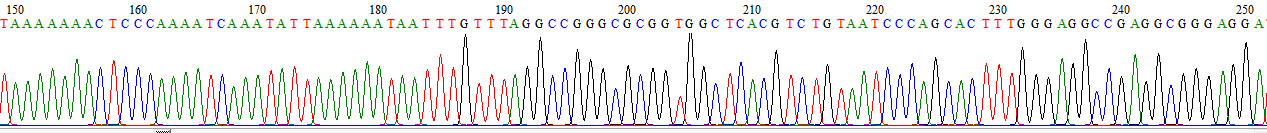


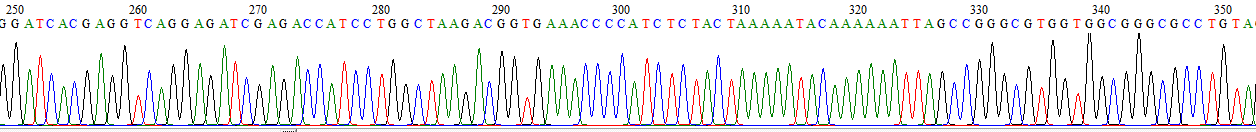


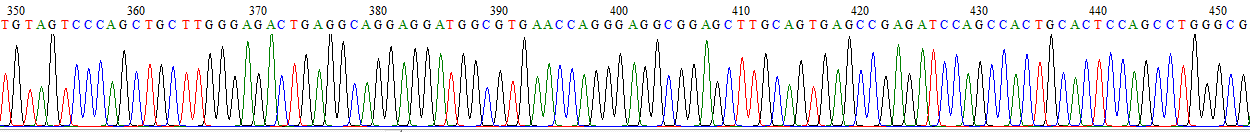


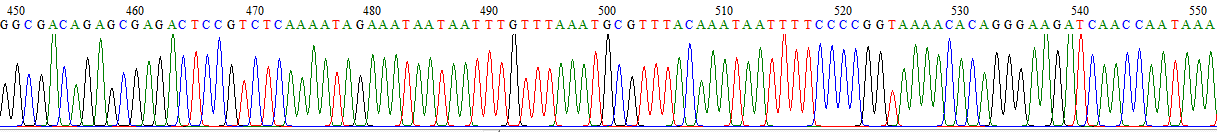


-1537C


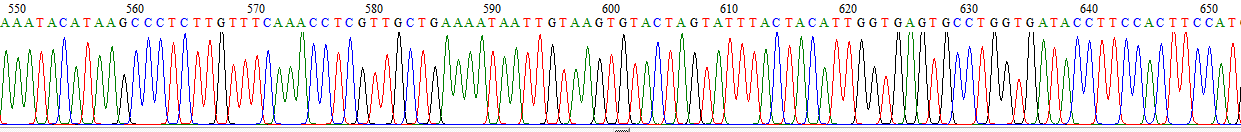


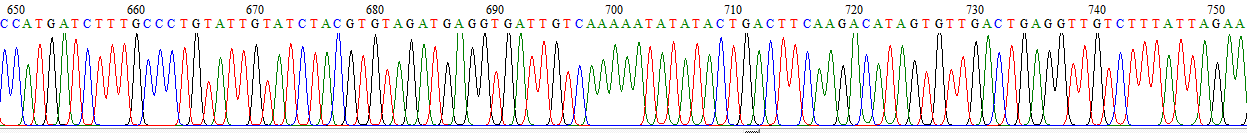


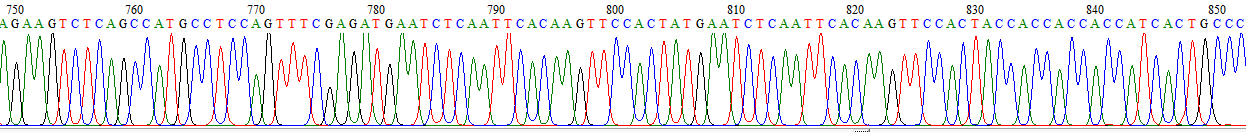


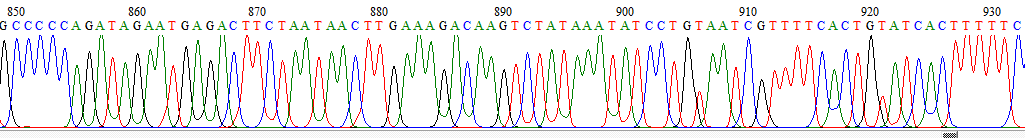


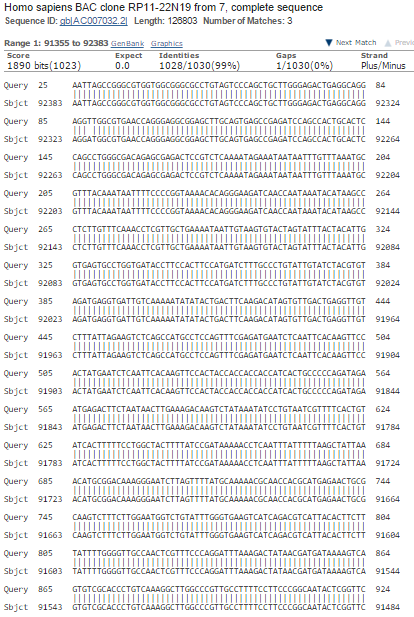


-1537C


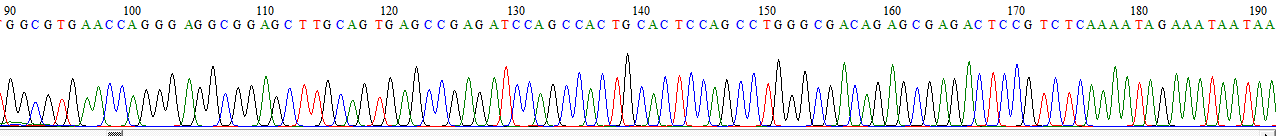


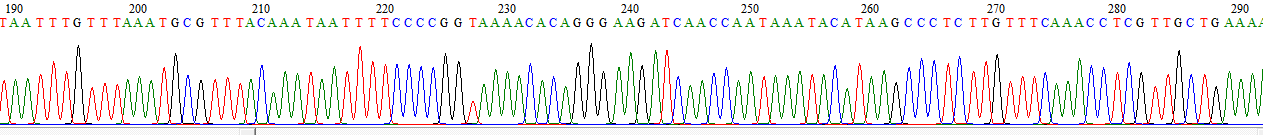


-1537C


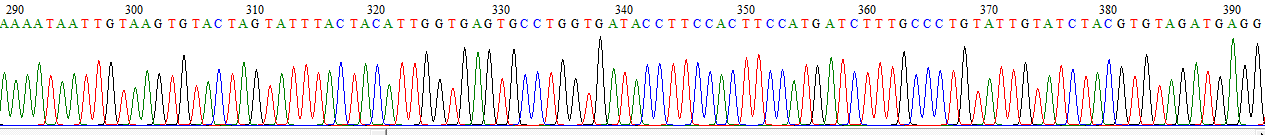


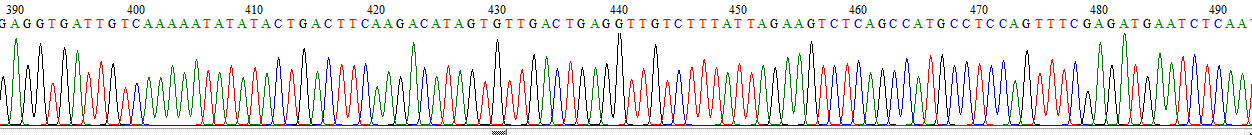


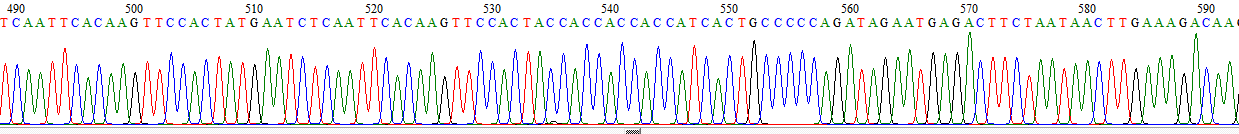


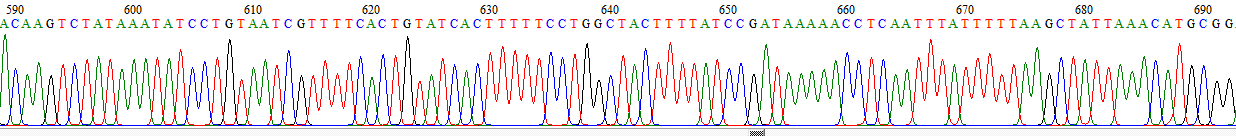


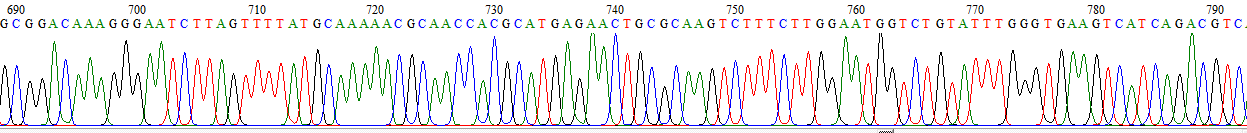


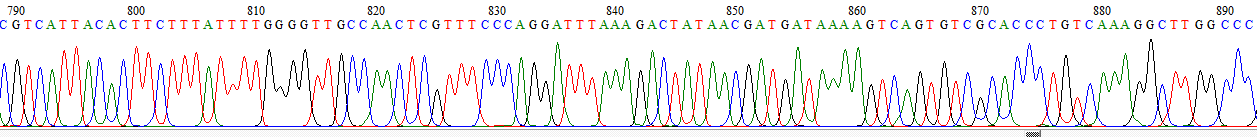


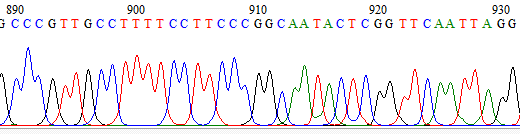


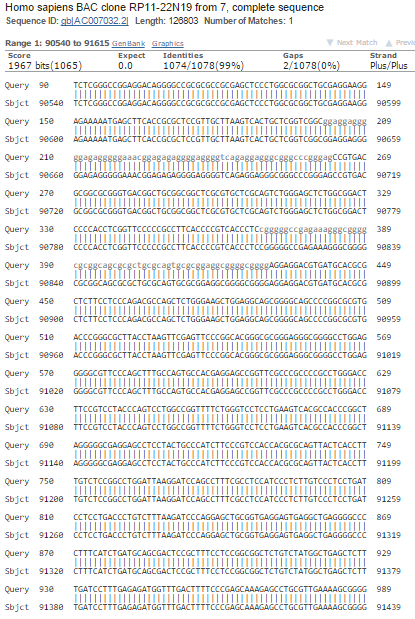


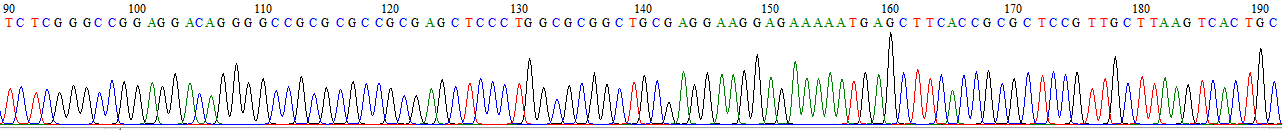


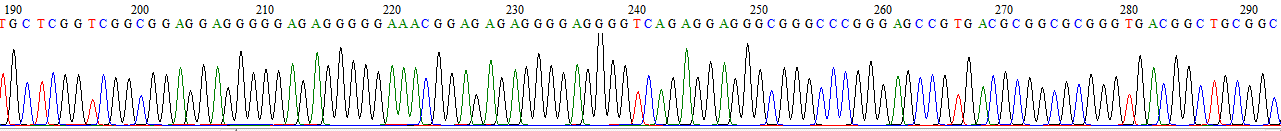


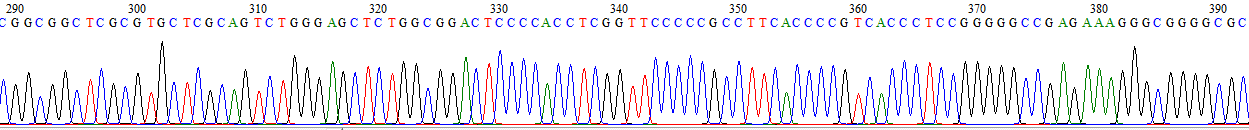


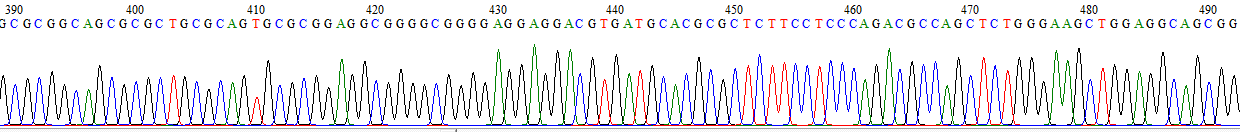


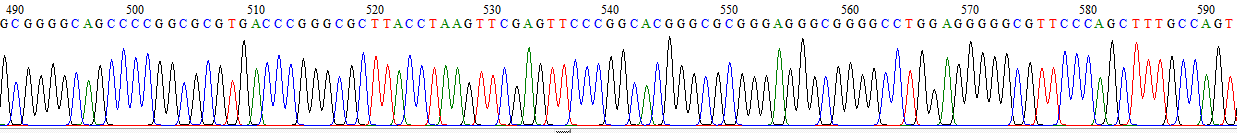


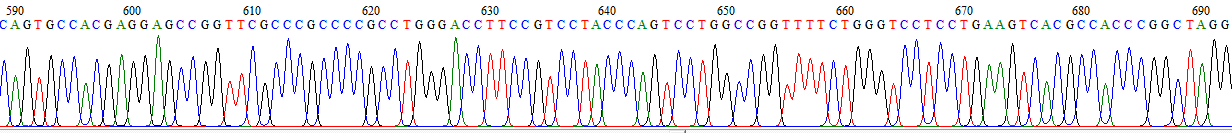


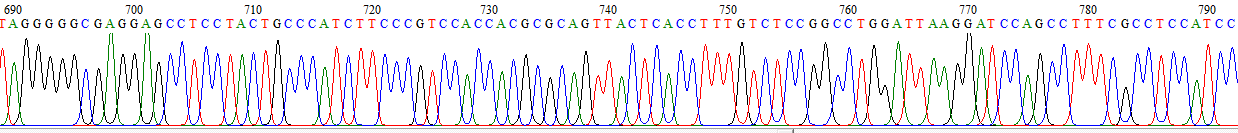


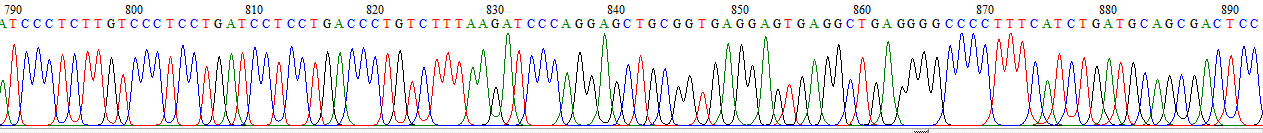


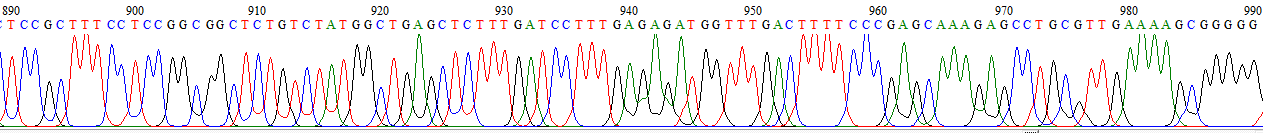

Supplement: Additional file 1: — Supplementary Figure 1. Plasmid sequence for wild-type visfatin promoter. (DOCX 867 kb) [file 12864_2016_3315_MOESM1_ESM.docx]

**Supplementary Figure 2**


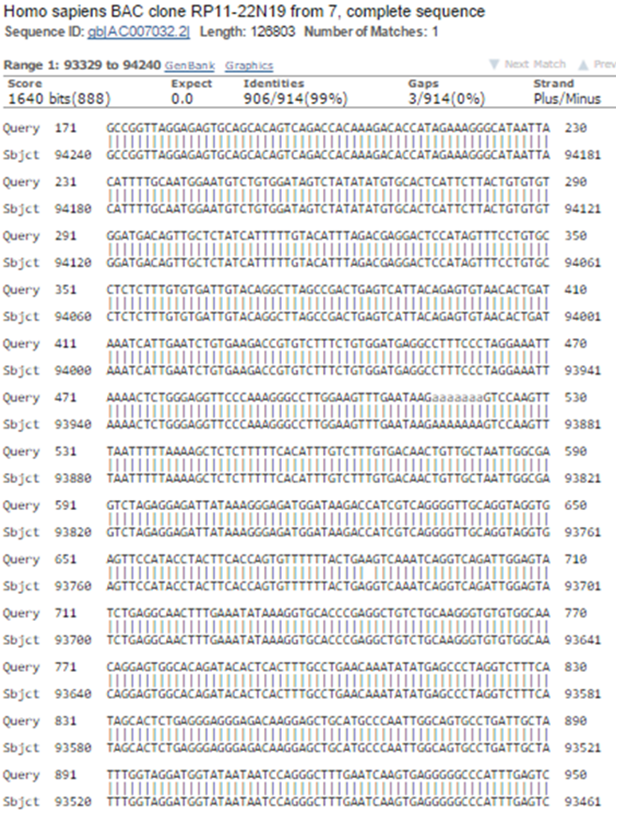


-3187G>A


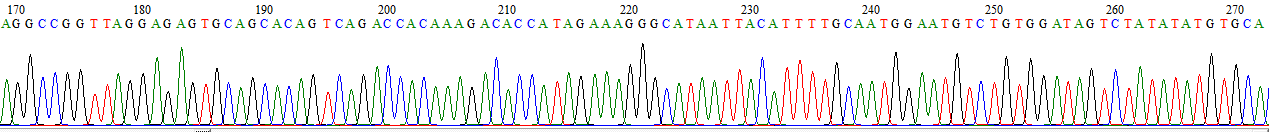


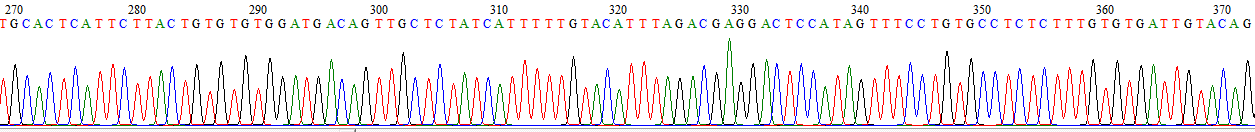


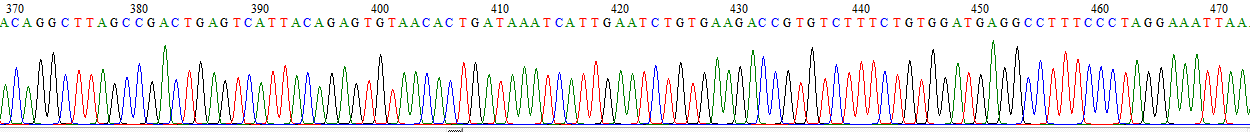


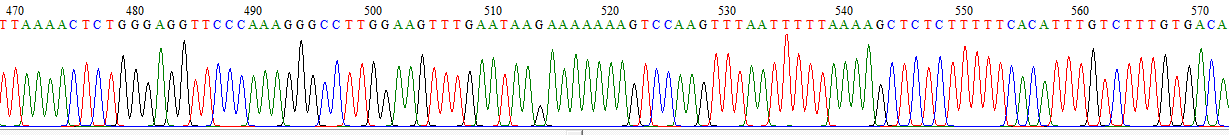


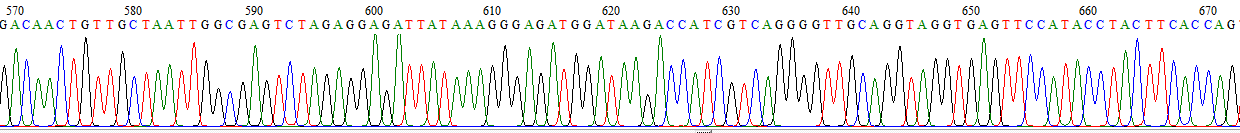


-3187G>A


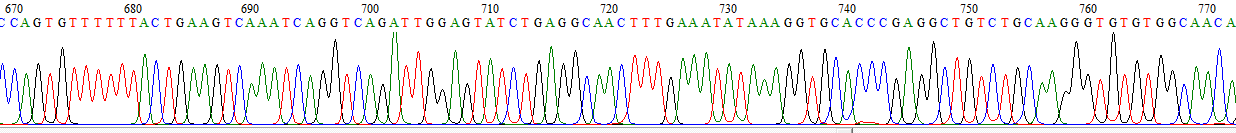


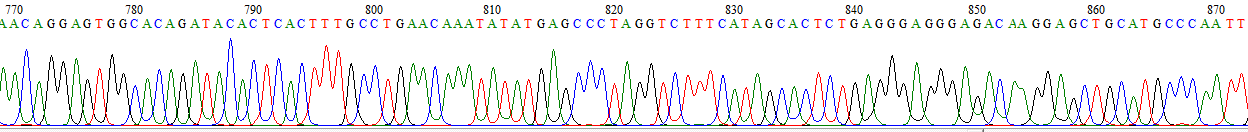


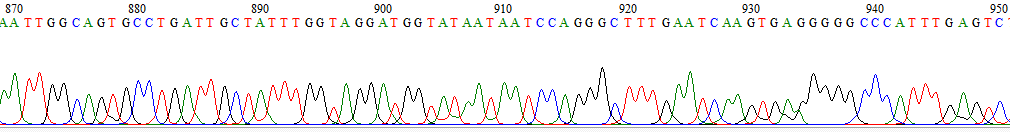


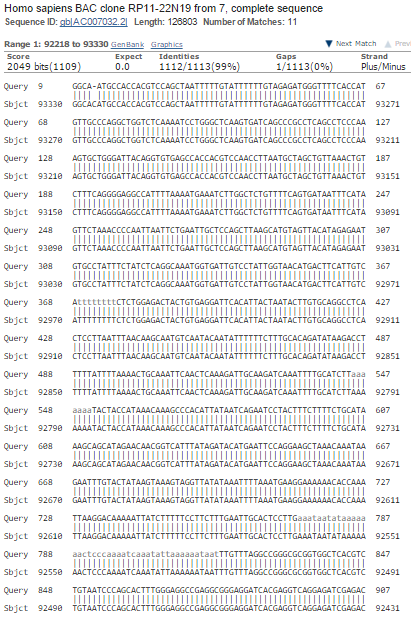


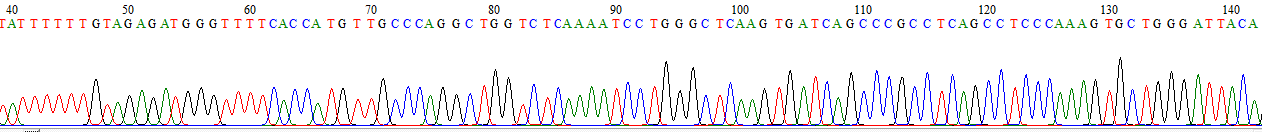


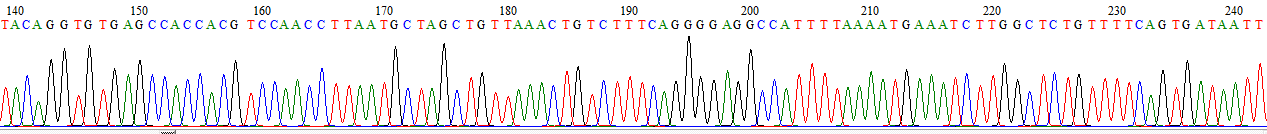


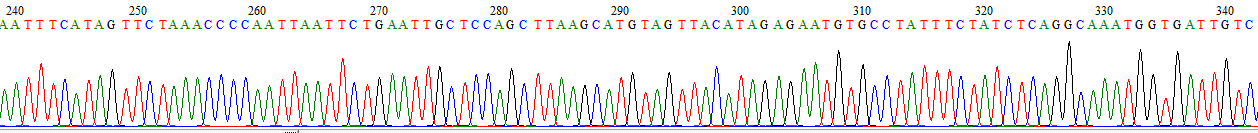


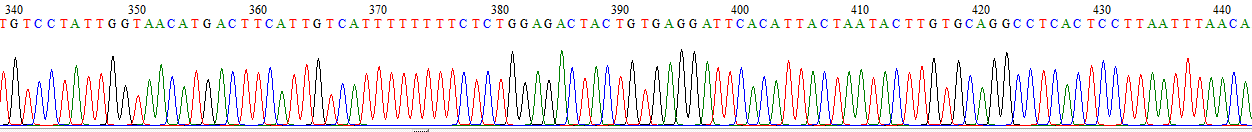


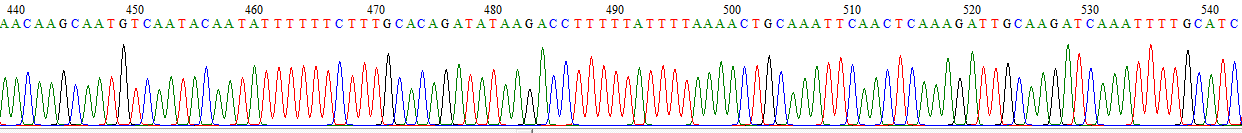


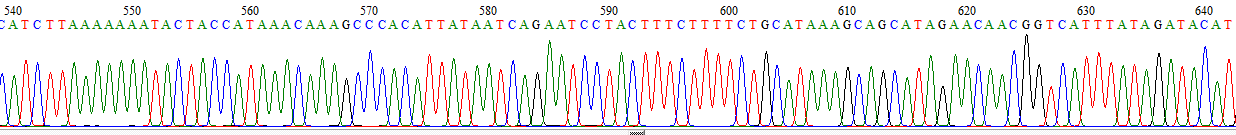


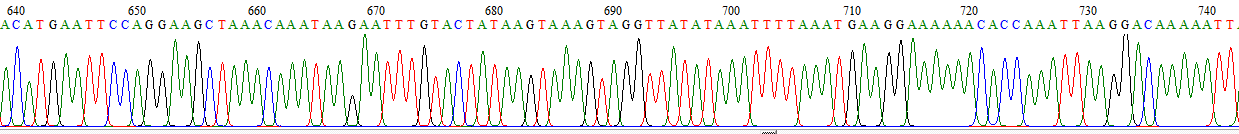


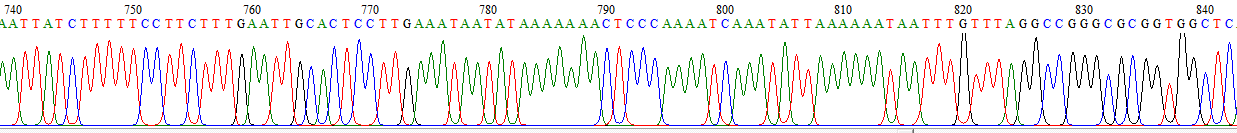


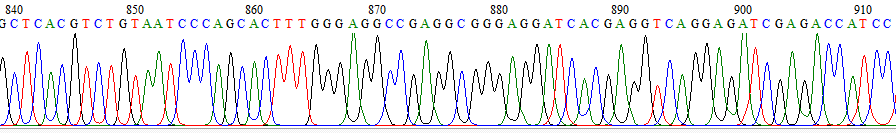


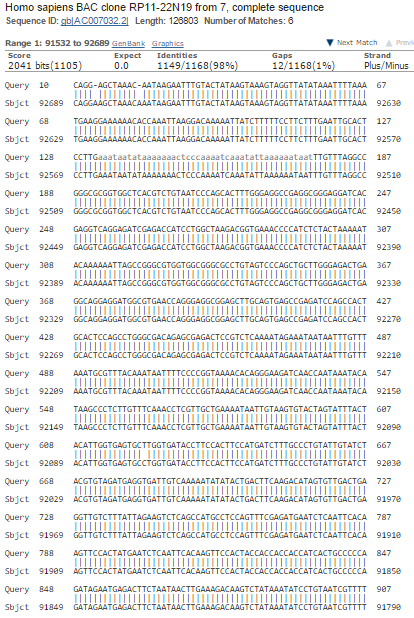


-1537C>T


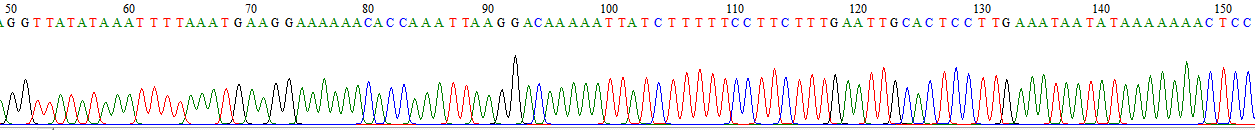


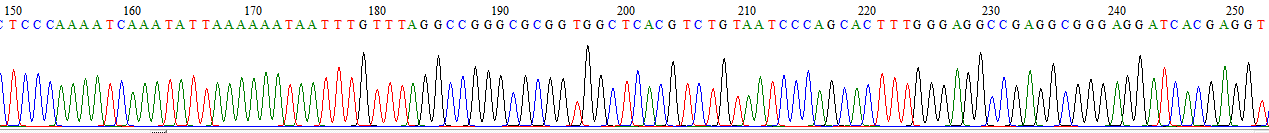


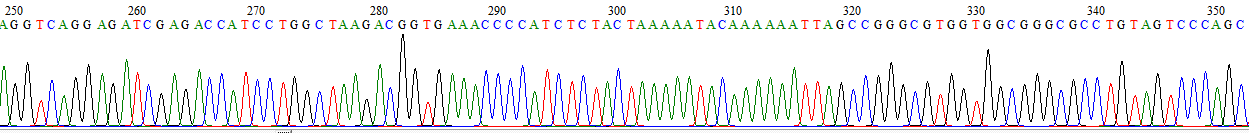


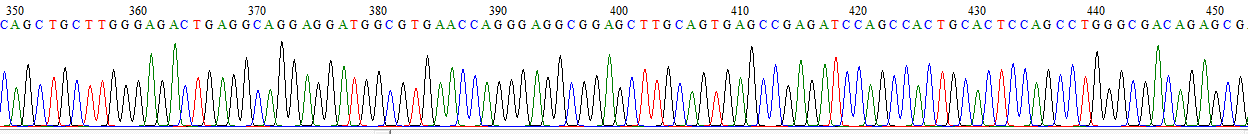


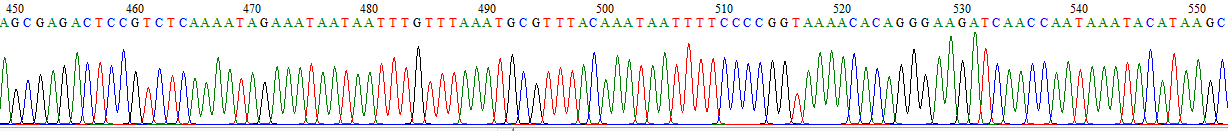


-1537C>T


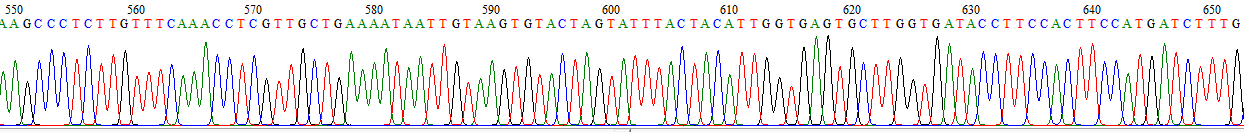


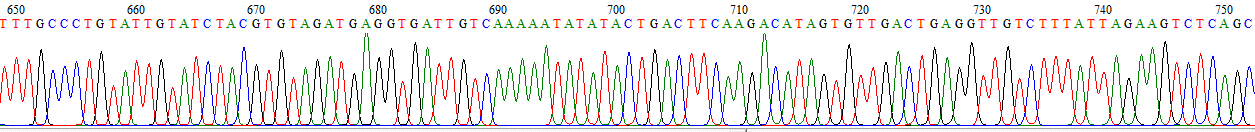


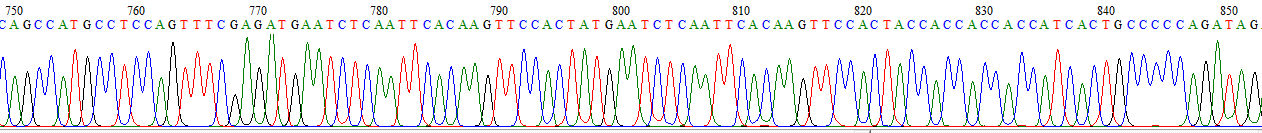


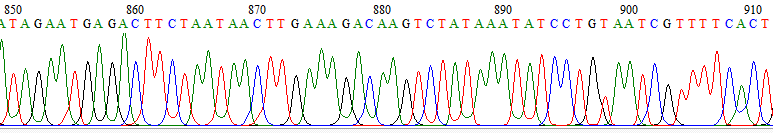


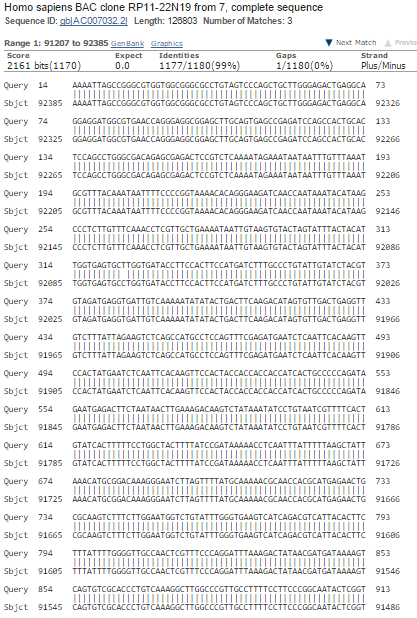


-1537C>T


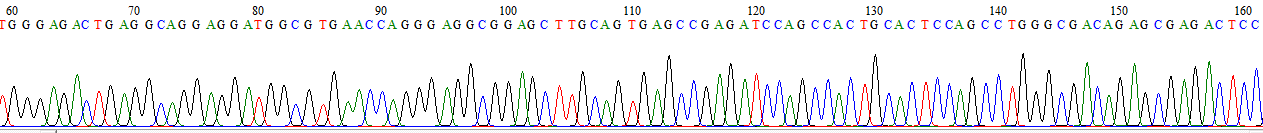


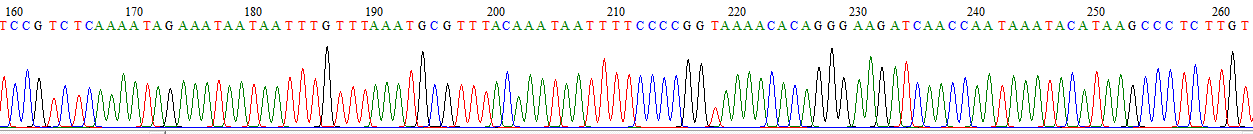


-1537C>T


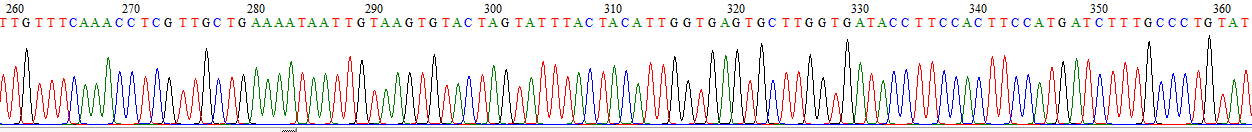


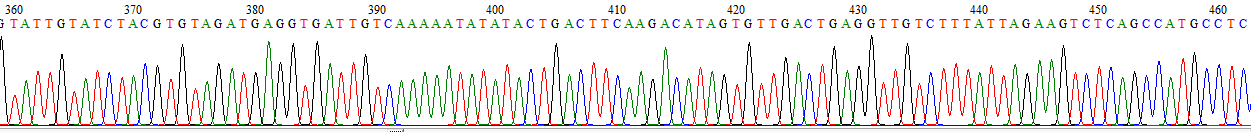


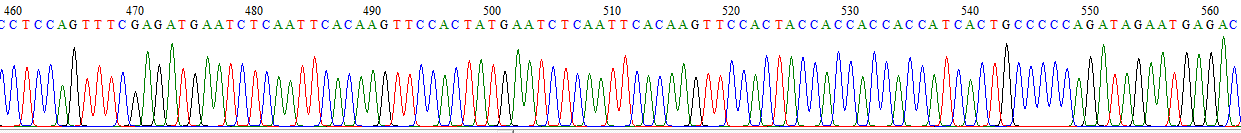


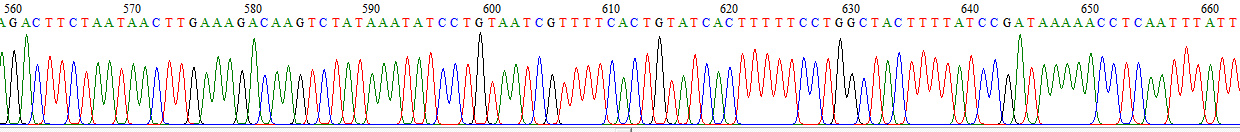


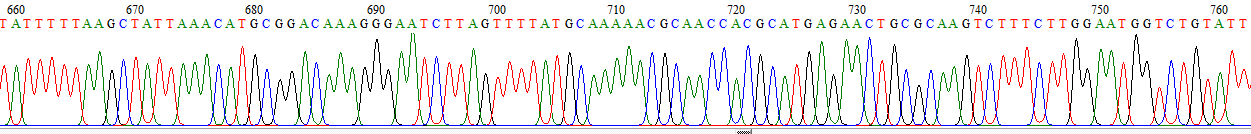


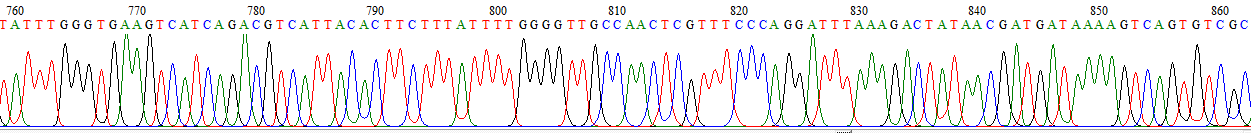


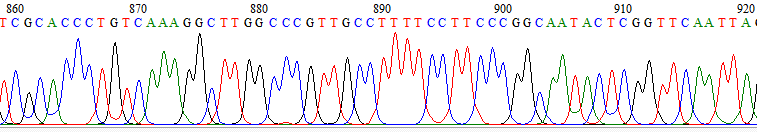


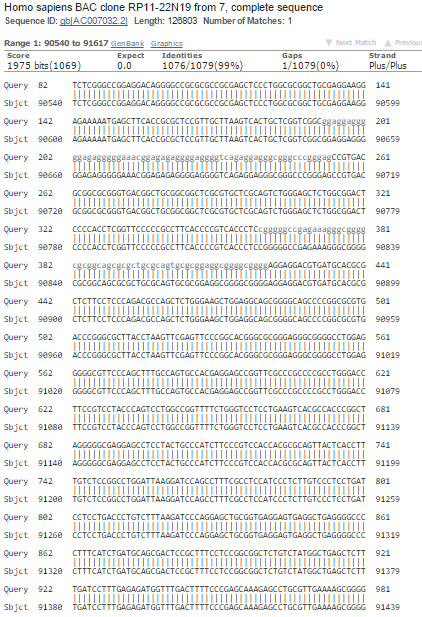


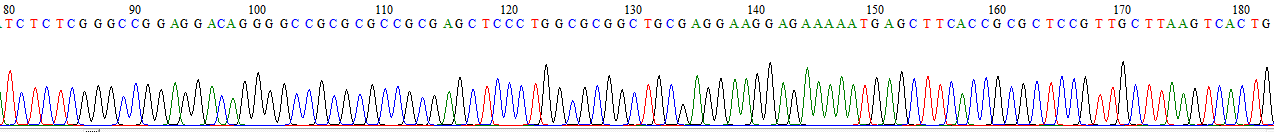


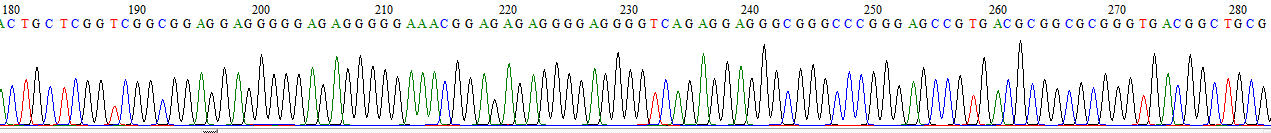


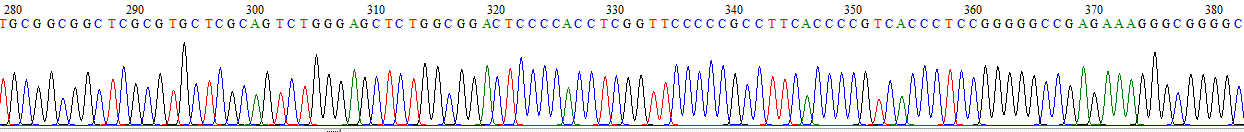


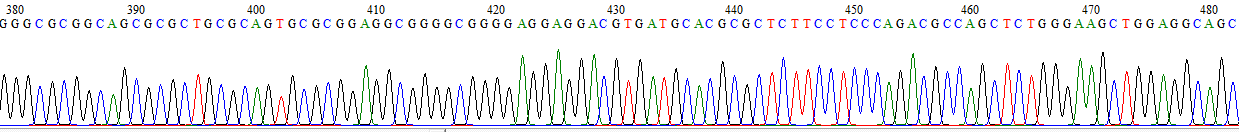


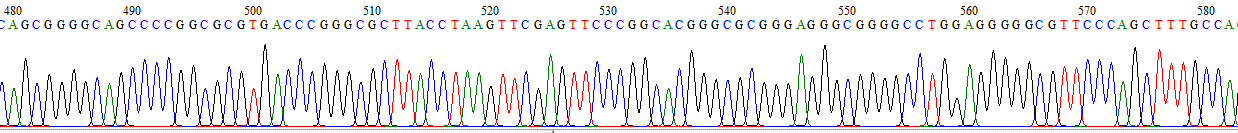


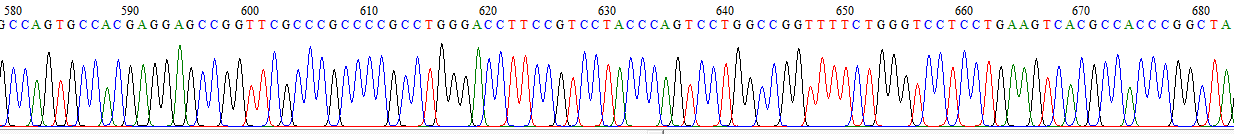


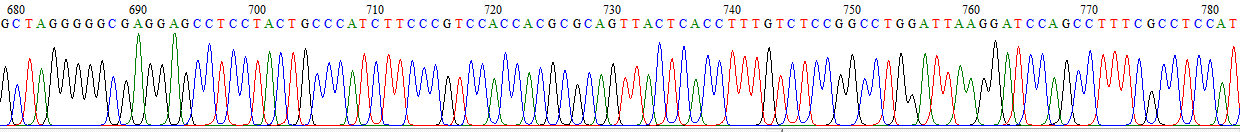


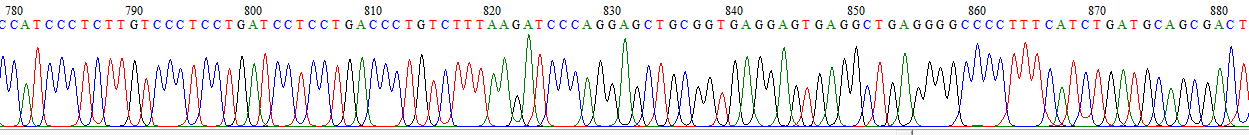


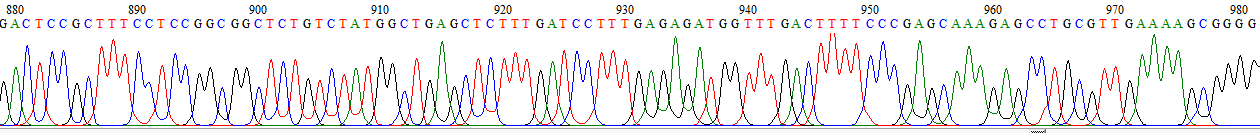

Supplement: Additional file 2: — Supplementary Figure 2. Plasmid sequence for variant visfatin promoter. (DOCX 1313 kb) [file 12864_2016_3315_MOESM2_ESM.docx]
