# Supplementary material for: In-vitro function of upstream visfatin polymorphisms that are associated with adverse cardiometabolic parameters in obese children
Source: BMC Genomics. 2016 Nov 25;17:974. doi: 10.1186/s12864-016-3315-9 (PMC5124300; doi:10.1186/s12864-016-3315-9)
Supplement: Additional file 3: — Supplementary Figure 3. ﻿Biological replicates for luciferase assays﻿. (DOCX 233 kb) [file 12864_2016_3315_MOESM3_ESM.docx]

**Supplementary Figure 3**

p=0.004

p=0.001

p=0.001

**A**

p=0.093

p=0.001

p=0.001

p=0.002

**B**

p=0.029

p=0.04

p=0.001

p=0.001

p=0.001

**C**

p=0.023

p=0.001

p=0.003

**D**

p=0.129
