# Supplementary material for: In-vitro function of upstream visfatin polymorphisms that are associated with adverse cardiometabolic parameters in obese children
Source: BMC Genomics. 2016 Nov 25;17:974. doi: 10.1186/s12864-016-3315-9 (PMC5124300; doi:10.1186/s12864-016-3315-9)
Supplement: Additional file 4: — Biological replicate for EMSA. (DOCX 372 kb) [file 12864_2016_3315_MOESM4_ESM.docx]

**Supplementary Figure 4**

**A**

**B**

1 2 3 4 5 6

1 2 3 4 5 6


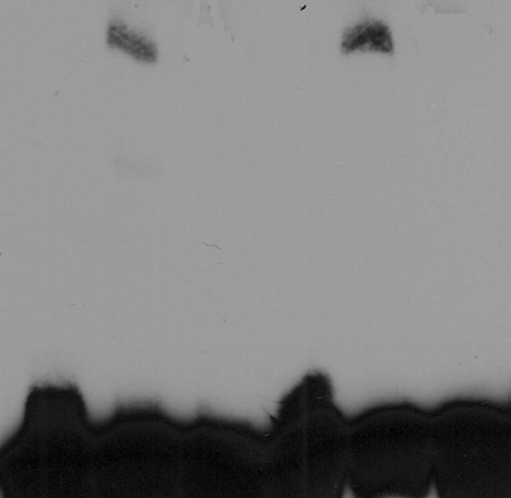

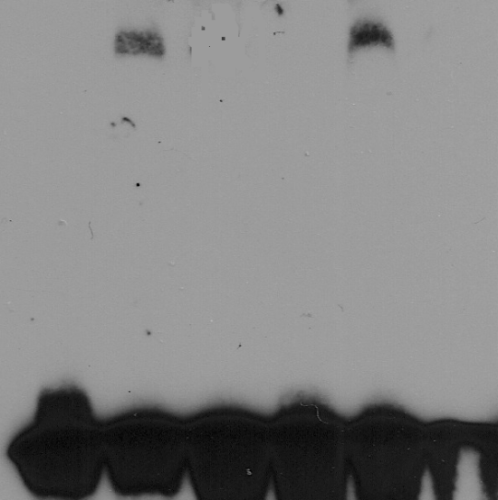


A: EMSA for c.-3187G>A SNP only, B: EMSA for c.-1537C>T SNP only, C: Assessment of binding between visfatin SNPs and nuclear protein. (1): Wild-type sequence without nuclear protein, (2): Wild-type sequence with nuclear protein, (3): Wild-type sequence with nuclear protein and unlabeled probe, (4): Variant sequence without nuclear protein, (5): Variant sequence with nuclear protein, (6): Variant sequence with nuclear protein and unlabeled probe
